# Joy in Movement: Traditional Sporting Games and Emotional Experience in Elementary Physical Education

**DOI:** 10.3389/fpsyg.2020.588640

**Published:** 2020-11-05

**Authors:** Verónica Alcaraz-Muñoz, María Isabel Cifo Izquierdo, Gemma Maria Gea García, José Ignacio Alonso Roque, Juan Luis Yuste Lucas

**Affiliations:** ^1^Faculty of Education, Murcia University, Murcia, Spain; ^2^Faculty of Sports, San Antonio Catholic University (UCAM), Murcia, Spain

**Keywords:** emotional well-being, traditional games, interpersonal relationships, emotions, physical education

## Abstract

Through games a motivating learning climate is provided, generating mainly positive emotions among the students by the very nature of the game. However, while the early stages are the most important for emotional well-being development, research about scientific knowledge of emotional physical education in children is still scarce. The aims of this study were to analyze the intensity of emotions (positive or negative) produced when players took part in games of different social structure, with or without competition (winner or loser), with or without sport experience and to examine the explanations given by the participants for these emotional experiences. Participants (*N* = 152) were recruited from two Spanish elementary school. We applied Student’s *t*-test and one-factor ANOVA. Students’ subjective comments were classified through content analysis in macro-categories and we used the Chi-square Automatic Interaction Detector (CHAID, implemented in SPSS^TM^ Answer Tree^®^ 13.0). The application of a mixed-methods approach identified statistically significant differences in four variables: (a) the type of emotion, (b) motor domain, (c) type of outcome (winning, losing, and non-competitive), and (d) sport experience. The intensity of positive emotions was higher (*M* = 3.71, SD = 0.893) than negative emotions (*M* = 1.18, SD = 0.253, *p* < 0.001). Furthermore, negative emotions were felt with different intensities (*F*_3_ = 3.82, *p* = 0.011, ES = 0.071), depending on the motor action domain. Comments referring to negative emotions were more frequent in individual games. Winning was associated significantly (*p* < 0.05) with the highest intensity ratings of positive emotions, whereas losing produced the highest values for negative emotions. The intensity ratings for positive or negative emotions not were different between non-competitive games and competitive games. The sport experience relativizes the mean of emotional intensity, both positive and negative. The present study brings the value of considering games as a key role to promote a physical education addressed to the education of social-emotional well-being in schoolchildren, as the basis of academic training. Furthermore, the results could benefit teachers as well as coaches have scientific input to organize teaching content, generating the desired motor behaviors together with positive experiences.

## Introduction

When a child is playing, he makes decisions, establishes social relationships, responds with motor actions and lives a unique emotional scenario. The interaction of each of these dimensions (cognitive, social, physical, and emotional) is what is known according to Parlebas (2018) as the “*pedagogy of motor behaviours*.” The mechanical concept of movement is replaced by motor behavior loaded of meaning. The motor skills are not the main reference, but instead the person who moves, making decisions, acting by emotional impulses, and interaction with other players (Lavega, 2018).

Each game has an internal logic that offers the player relations with other players, space, material, and time. The emotional experience is conditioned by the very nature of the game, offering various motor behaviors (Shahid et al., 2008). The players’ characteristics, their fears, worries, hobbies, or sport experience (Gea et al., 2016), understood as the external logic of the game, will also influence their emotions (Alcaraz-Muñoz et al., 2017) and even their degree of enjoyment of the game (García et al., 2010). In the same game, the players act differently, for example, in hide-and-seek, there are children who prefer to remain hidden until the last moment to avoid being caught, whereas others, on the contrary, more enjoy assuming the risk of being caught.

In motor praxeology, Parlebas (2001) proposed the domains of motor action to classify the games according to the social relationships between players: (a) the psychomotor domain, in which the player acts by himself in the game, without any motor communication with others, such as when playing with a spinning top or hopscotch; (b) the cooperation domain, in which players jointly participate to achieve the game’s goal among all of them, such as a relay race or the parachute game; (c) the opposition domain, in which players only act as adversaries to prevent the rest of the players from achieving the motor objective, as in black and white or arm wrestling; (d) The cooperation-opposition domain, which combines opposition between rivals with collaboration between teammates, as in the prisoner’s ball or the ultimate.

When choosing a game, another relevant aspect to be analyzed due to its influence on the players’ emotional experience is the way it ends (Harvey and O’Donovan, 2013; Serna et al., 2017). When referring to games that end when they fulfill an inherent purpose determined by the rules of the game (internal logic), we imply competition games. On the contrary, when games stop due to some aspect that is external to the rules of the game, such the players’ decision, the atmospheric weather, snack time, etc. (external logic), we are referring to games without competition (Suits, 1978).

According to Lazarus (1991) and Bisquerra (2003), the emotions experienced can be classified as positive or negative. Positive emotions, such as joy, happiness, good humor, and love (understood as affection) are experienced in situations of well-being, whereas negative emotions such as anger, sadness, rejection, fear, and shame, are triggered in situations of discomfort. The same game is a different emotional scenario for each player, depending on the type of emotion felt, the degree of intensity experienced, his or her personality, preferences, fears, interactions with other players, and even the rules of the game itself (Frijda, 2007; Diener et al., 2009). For example, in the game of black and white (opposition with competition), each player can experience joy or sadness, depending on whether he has won by catching his opponent more times or has lost for being the most frequently caught player. In the game of steal-the-hanky (opposition without competition), the person who manages to catch the handkerchief of an adversary will feel joy, whereas the person who has lost the handkerchief may feel sad. Emotions, whether they are triggered by favorable or unfavorable situations, play a very important role in people’s motor intervention.

From the educational perspective, the most important thing is for teachers to achieve an adequate relationship between the educational objectives and the proposed games. Understanding this relationship allows them to reflect on their practice, make decisions and propose games based on empirical evidence to produce different emotional experiences in their students depending on the type of game (Parlebas, 2001; Puig and Vilanova, 2011). In addition, these types of motor experiences not only stimulate students’ development on a cognitive and physical level, but also on a social and emotional level (Bailey et al., 2009). The fact of participating in games arouses different emotions that must be recognized by the players, thus increasing their emotional awareness, a key aspect for the development of social skills and other emotional competencies (Bisquerra, 2003; Laborde et al., 2015; Zysberg and Hemmel, 2017). In this sense, physical education plays a key role because of its inseparable relationship between games and emotions (Lavega et al., 2014; Cañabate et al., 2018; Duran and Costes, 2018). In addition, regarding children’s psycho-evolutionary and social development, the stage from 6 to 12 years is the period in which the capacity to acquire personal autonomy develops, and intrapersonal and interpersonal relations increase. Thus, it is essential for physical education teachers to have scientific knowledge of emotional physical education from early stages. We need to teach children to know and understand their own emotions and those of others (Shahid et al., 2008).

Through games, a motivating learning climate is provided, generating mainly positive emotions among the students (Light, 2008; Howard et al., 2017). Positive emotions have always been rated with higher intensity in the different domains of motor action (Lavega et al., 2014; Miralles et al., 2017; Duran and Costes, 2018). Sociomotor games present a higher emotional intensity, especially cooperative games where common challenges are established and pacts or negotiations with the other players are required (Fredrickson, 2001; Desivilya and Yagil, 2005; Waugh and Fredrickson, 2006), followed by the cooperation-opposition games and lastly, by opposition games. The presence or absence of competition is another factor to consider in the intensity of the emotional experience. Negative emotions are more intense than positive ones when there is a possibility of finishing the game as the winner or the loser, mainly due to the increase of conflicts or fouls during the games (Helmsen et al., 2012).

Based on these theoretical arguments, the present research proposed two main objectives: (a) to analyze the intensity of the emotions (positive or negative) felt by the players in different domains of motor action (psychomotor, cooperation, opposition, and cooperation-opposition); in non-competitive games, and in competitive games when they are winners or losers; with or without sport experience and (b) to examine the players’ comments about the most intense emotions experienced in the four domains. From these objectives the following hypothesis are extracted: (1) traditional sporting games encourage the experience of positive emotions; (2) each domain of motor action generates a certain type of emotions in the players; (3) the presence of competition increases the emotional intensity experienced by schoolchildren; and even more positive emotions if they are winners and negative emotions when they lose; and (4) when players have sports experience, they experience emotions with greater intensity.

## Materials and Methods

### Design and Participants

A descriptive and cross-sectional study design with on-probability-based sampling was used. The participants were 152 students of Elementary Education (72 boys and 80 girls; age range = 8–12 years, *M*_*age*_ = 9.72, SD = 1.18), belonging to two Spanish schools. Of them, 41 participants practiced sports in federated clubs at least three times a week, 63 performed physical activity once or twice a week and 48 participants did not perform physical activity. The fathers, mothers, and/or legal guardians of the children—all minors—gave their consent to participate in the study, which was also approved by the University’s Research Ethics Committee.

### Measurements and Materials

#### Games and Emotions Scale for Children Questionnaire

The intensity of the emotions experienced in each game was assessed by the Games and Emotions Scale for Children (GESC), validated by Alcaraz-Muñoz et al. (in review). This version of the GESC questionnaire used had a total of nine items, which were grouped in two factors of emotions identified as: positive emotions (four items: joy, humor, affection, and happiness) and negative emotions (five items: sadness, fear, anger, rejection, and shame) (Lazarus, 1991; Bisquerra, 2003). Each of these items related with the two factors of emotions was measured through a Likert-type scale ranging from 0 (*I did not feel anything*) to 4 (*I felt a lot*). For each identified emotion (positive or negative) a mean value of emotional intensity experienced was obtained.

In terms of reliability, the Cronbach alpha value indicated that the internal consistency was good both for positive (α = 0.85) and negative (α = 0.77) emotions. Confirmatory factorial analysis adequately reproduced the scale structure and showed good fit indices [minimum χ^2^/df = 1.35; Tucker-Lewis index (TLI) = 0.98; incremental fit index (IFI) = 0.98; comparative fit index (CFI) = 0.98; mean quadratic approximation error (RMSEA) = 0.048 (LO90 = 0.000 – HI90 = 0.086)].

#### Selection and Application of Games

Four 1-hsessions were conducted by the teachers in charge of Physical Education at each school in the same order. The games used (Table 1) were selected based on the following criteria: (a) they were well-known games within the Spanish culture; (b) the games represented the four domains of motor action; (c) each session was dedicated to the domain of motor action, combining a non-competitive and a competitive game (with winners and losers) in all sessions in the same way; (d) the habitual practice carried out by schoolchildren in their real Physical Education context was respected; and (e) the pedagogical principles for the training of the students in games were maintained, such as the equal participation of all the students. The sequencing of the sessions was established following an increasing order of decision complexity determined by the motor action domains of each game: psychomotor games, cooperation games, opposition games, cooperation-opposition games.

**TABLE 1:** Description of the games used in the study.

**Type of game**		**Description**
Emotional awareness games	Dramatization game	Nine children are chosen randomly and assigned one of the nine emotions without them knowing what it is. Once the emotion is assigned, they will be given a card, which they will hold behind them (without it being seen by them at any time). These players will be placed distributed throughout the playing space. The rest of the participants, grouped in pairs, must go through each area and mimic the emotion that the child who occupies that place is supposed to represent. At the end, the child who does not know the emotion should name aloud the emotion that he thinks it represents and why he believes it.
	Stealing-stone	The playing field is divided into two equal parts. In each field a team is placed with its respective stones (balls or similar material) placed at the bottom of the field of play. The purpose is to accumulate the largest number of stones. To do this, teams can choose to: (a) place players in their own field, which makes them defenders. Its function is to prevent the opposing team from stealing stones, for this the mechanics of a tag game will be followed, so that when touching a rival player he must be placed in the same area of the stones, being able to be saved if a teammate crashes their hands with it, or (b) the players can choose to go to the opposite field, which makes them attackers, their main function being to try to steal a stone or free a caught teammate. At the end of the game, the number of stones that each team has obtained is counted, giving the victory to the one with the highest accumulated number of stones.
Psychomotor games	Jump, jump, little lobster (non-competitive)	Each player has a jumping rope for free-form jumping. When the teacher claps, the players must jump forward, two claps, they should jump back, and three claps, they jump on one leg.
	Quick kangaroo (competitive)	In pairs. The players compete with each other trying to achieve the highest number of rope-jumps. In the second part, the players occupy parallel zones to race while rope-jumping. The general winner is the player who gets the most wins per round.
Cooperation games	Pass and receive (non-competitive)	Teams of five players in circles. Each team tries to achieve the objective of the game by improving their score each time. Players must pass a ball to each other, dribbling once on the ground, and avoid passing it to the classmate on their right or left side. The goal is to achieve the highest number of passes. To increase the difficulty, a second ball is used.
	Pass and win (competitive)	The dynamics of the previous game are maintained, but the results between teams are now compared. The winning team is the one that manages to make more passes per rounds.
Opposition games	Steal-the-hanky (non-competitive)	The players stand in two facing rows, located at a distance of 10 m. Each player is assigned a random number depending on the number of players per row. The teacher stands in the center of the two rows holding a handkerchief. The teacher says a number aloud and the players of the two rows that have that number assigned to them run out to grab the handkerchief. The player who manages to grab it must return to his or her row without being caught by the other player.
	Black and white (competitive)	In pairs, face to face, situated 1 m from each other. Each player is assigned a name (black or white). The teacher says one of the two names at random. The named child tries to catch the adversary before he or she reaches the end of the track. The winner is the one who catches the other player or escapes without being caught more times.
Cooperation-opposition games	Killing ball (non-competitive)	Players are located within a 15 × 15 m space. One player moves out of this space with a ball. The goal is to try to hit the other players with that ball. The player who is hit must leave the delimited space and join the hitter. The game ends when there are no players left in the space.
	Square mate (competitive)	Two teams. The team playing the zombie role is located within a delimited space. The players of the other team (the hunters) move around outside of this space, passing a ball to each other to try to hit the zombies. The hit zombies remain seated while trying to touch the ball to rejoin the zombies. The teams change the roles after the set time, at the end of that time, the seated zombies are counted. The winning team is the one who manages to seat more zombies.

Finally, when classifying each of the games, their categorization was taken into consideration based on the possibility or not of obtaining victory within the practical motor situation developed (Parlebas, 2001). This allows to differentiate between two types of games: (a) games with competition or competitive games, which are those traditional motor games that are identified with the presence of a final score or result within the game, which allows the player to obtain or not victory at the end of the practical situation, and therefore, be classified as a winning or losing player; (b) games without competition or non-competitive games, which are those traditional motor games that do not have a final score, and therefore, there is no final that classifies the players based on obtaining victory or not within motor practice, not being its base the competition (Etxebeste et al., 2014; Gea et al., 2016).

### Procedures

#### Initial Familiarization: Students’ Emotional Awareness

Students participated in a 1-h session, which provided them with theoretical and practical knowledge about emotions based on the models of Lazarus (1991) and Bisquerra (2003). In this session, the children learned how to identify their own emotions, first, by commenting on everyday situations where they had noticed emotions such as joy, happiness, affection, humor, sadness, anger, fear, shame, and rejection. To facilitate the correct identification of emotions, a theoretical introduction was made with examples of each emotion and a simulated daily life situation (for example, “*on your birthday, you receive the toy you wanted as a gift, the possible emotion associated with this situation being happiness*” or “*You are playing with your favorite toy when suddenly it falls and breaks, possibly triggering an emotion of sadness*”). Secondly, in order to extrapolate the identification of emotions to the situations of motor game, they participated in a game focused on the dramatization of these emotions, and then in the game of stealing-stones (cooperation-opposition with competition) to simulate the situations of games of the rest of sessions (Table 1). At the end of this game, they were asked to describe a specific situation of the game in which they had identified one of the emotions that had been explained earlier, for example, joy when stealing a stone from the opposing team, sadness when they were caught, etc. The students were also taught how to respond on the scale of emotional experience expression.

#### Measures

Four practical sessions were developed corresponding to the four motor action domains. Although the participants were already familiar with the questionnaire, the teachers proceeded to briefly recall at the beginning of each of the sessions the structure and sections to fill out. At the end of each game, the students marked on their personal copy of the GESC the level of intensity (from 0 to 4) that they had felt for each of the nine basic emotions enumerated (Lazarus, 1991; Bisquerra, 2003), according to the graphic representations of the degree of intensity of each emotion. Immediately afterward, they were asked to write a brief explanation of why they thought they had felt the most intense emotion, and to draw the aspect of the game that they had found most striking, with a pleasant and/or unpleasant connotation.

### Statistical Analysis

#### Quantitative Data Analysis

Descriptive data are presented as means and standard deviation of the mean. The Kolmogorov–Smirnov test was used to test for normality. All the data had a homogeneous distribution. Hence, we applied parametric tests: Student’s *t*-test for independent samples and related samples with categorical variables with two groups, and one-factor repeated measures ANOVA for categorical variables with more than one group. A Bonferroni *post hoc* test was used to explore the differences among the conditions. Greenhouse–Geisser correction was used to check the sphericity assumption. We used a *p*-value of 0.05 for all statistical tests. We also calculated the effect size (ES) results of the interactions between the variables, using partial eta square (ηP^2^) and Cohen’s *d* [0.2 (small); 0.5 (medium), and >0.8 (large) effect]. All analyses were performed using the Statistical Package for Social Sciences version 22.0 for Windows.

When examining the data analysis, the correlations between variables were: (a) type of emotion (positive or negative), (b) type of motor action domain (psychomotor, cooperation, opposition, and cooperation-opposition), and type of game (not competitive or competitive). For independent samples, the correlations were: (a) type of outcome (winning or losing), and (b) type of emotion (positive or negative), and (c) type of motor action domain (psychomotor, cooperation, opposition, and cooperation-opposition). Intra-subject relationships were: (a) type of emotion (positive or negative), and (b) type of motor action domain (psychomotor, cooperation, opposition, and cooperation-opposition).

#### Qualitative Data Analysis

Students’ subjective comments about the most intense emotion were firstly classified through content analysis in macro-categories: aspects referring to the internal logic and aspects related to the external logic of the game. Next, we analyzed the presence or absence of micro-categories referring to the internal or external logic.

Internal logic: (A) internal time: allusions to the end of the game “*winning*,” “*losing*,” or to “*competition*.” In games without competition, comments on the duration or the objective of the game, changes of role (e.g., “*It is fun to switch from hare to hunter*”); (b) internal space: terms relating to the play field or more generic comments on the positions of the body in space and their postures (e.g., “*It was difficult to run while jumping*”); (c) internal relationship: comments on the motor interaction with other players (e.g., “*My classmates passed me the ball*”); (d) internal material: allusions to the objects (ball, handkerchief, rope) (e.g., “*Hitting with the ball was difficult*”); (e) rules: presence or absence of terms about the agreements (e.g., “*The rules are complicated*”) or the game in general (e.g., “*The game is fun*”).

External logic. (a) External time: comments on temporal aspects external to the rules of the game (e.g., “*It was very early*”); (b) external space: terms relating to the facility or play field (e.g., “*The sports hall was dark*”); (c) external relationship: allusions to permanent personal attributions (e.g., “*I was playing with my friend*”); (d) external material: comments on the type of material used for the elaboration of the objects (e.g., “*The ball was hard*”); (e) persons (external): allusions to people’s transient states (e.g., “*We laughed a lot*”) (Lagardera et al., 2018).

A coding manual was developed for observers that included (a) the category system, (b) the code for each category, and (c) several examples. Six observers completed a minimum of 40 h of training on how to code the data, following the guidelines of Muñoz et al. (2018). To assess the reliability of the data, each of the six observers analyzed 100 comments. These data were compared, and inter-observer reliability was measured using the Kappa Index, which yielded values ranging from 0.78 to 0.84.

Using data obtained, descriptive statistical tests were performed to determine the distribution of frequencies of the variables. In parallel, a statistical model of a comprehensive classification tree (Morgan and Sonquist, 1963) was applied. Classification trees are a freely distributed procedure for conducting exploratory analyses, which show the relationship between variables according to different levels of importance. We used the Chi-square Automatic Interaction Detector (CHAID, implemented in SPSS^TM^ Answer Tree^®^ 13.0) classification tree method because it can build non-binary trees, that is, trees that can include more than two branches or divisions of data on each node, depending on the categories to be explained.

A comprehensive classification tree was generated to examine the predictive power of the different variables of the children’s comments about the game (winning, losing, and not competing) for the motor action domain (psychomotor, cooperation, opposition, and cooperation-opposition), according to the criterion of their presence or absence.

## Results

The results derive from three relevant interactions: (a) type of emotion and type of motor action domain; (b) type of emotion, type of outcome (winning, losing, or non-competitive game), and type of motor action domain; and (c) type of emotion and sport experience.

### Type of Emotion X Domains of Motor Action

**FIGURE 1:**
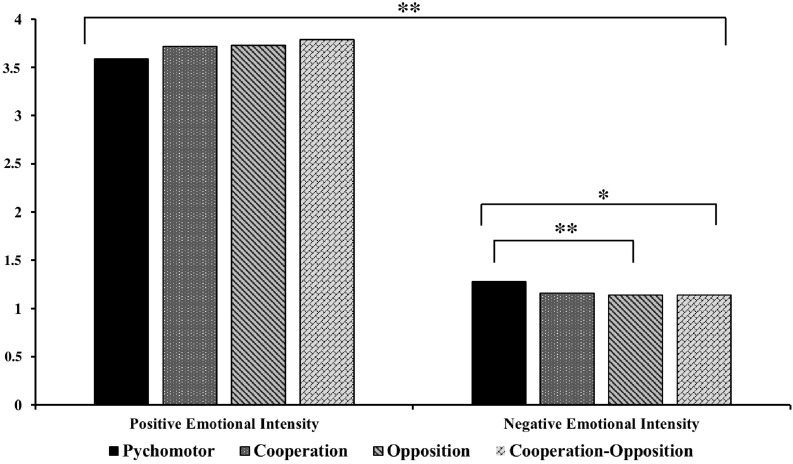
Positive and negative emotional intensity by motor-action domain. Data as shown as mean. ^∗∗^*p* < 0.01; ^∗^*p* < 0.05.

Student’s *t*-test for related samples showed that positive emotions scores obtained higher intensity (*M* = 3.71, SD = 0.893; *p* < 0.001) than negative emotions (*M* = 1.18, SD = 0.253) (*t* = 32.16, *p* < 0.001, ES = 2.61). The Type of emotion X Motor-action domain interaction showed that positive emotions were experienced with the same high intensity (*F*_3_ = 1.83, *p* = 0.114, ES = 0.015) in all four domains of motor action: psychomotor (*M* = 3.59, SD = 1.128), cooperation (*M* = 3.72, SD = 1.065), opposition (*M* = 3.73, SD = 1.043), and cooperation-opposition (*M* = 3.79, SD = 1.057) (Figure 1).

The results of the intra-subject relations showed that the negative emotions were felt with different intensities (*F*_3_ = 3.82, *p* = 0.011, ES = 0.071), depending on the motor action domain. The intensities in the psychomotor games were significantly higher (*M* = 1.28, SD = 0.006) than those of the opposition games (*M* = 1.14, SD = 0.293, *p* = 0.05) and the cooperation-opposition games (*M* = 1.14, SD = 0.298, *p* = 0.012), whereas no significant differences were found with the cooperation games (*M* = 1.16, SD = 0.358, *p* = 0.051) (Figure 1).

### Type of Emotion X Type of Outcome X Domains of Motor Action

With regard to the interaction between type of emotion, type of outcome, and type of motor action domain, in competition games, Student’s *t*-tests for independent samples showed that winning produced significantly higher scores for positive emotions in all four domains of motor action: psychomotor (*M* = 4.05, SD = 0.998, *p* = 0.000), cooperation (*M* = 4.18, SD = 0.999, *p* = 0.000), opposition (*M* = 3.92, SD = 1.094, *p* = 0.049), and cooperation-opposition games (*M* = 3.98, SD = 1.100, *p* = 0.013). Losing was associated with significantly higher scores for negative emotions in the case of psychomotor (*M* = 1.49, SD = 0.784, *p* = 0.005), cooperation (*M* = 1.25, SD = 0.490, *p* = 0.010), and cooperation-opposition games (*M* = 1.31, SD = 0.583, *p* = 0.002), whereas in opposition games, the level of intensity of negative emotions was similar, without significant differences either for winning (*M* = 1.10, SD = 0.339, *p* = 0.265) or losing (*M* = 1.17, SD = 0.468, ES = 0.17) (Table 2).

**TABLE 2:** Mean emotional intensities according to type of emotion, outcome, and motor action domain.

**Outcome**	**Motor action domain**	**Type of emotion**			
		**Positive**		**Negative**	
		**Mean**	**SD**	**Mean**	**SD**
Winning	Psychomotor	4.05	0.998	1.18	0.466
	Cooperation	4.18	0.999	1.08	0.271
	Opposition	3.92	1.094	1.10	0.339
	Cooperation-opposition	3.98	1.100	1.06	0.214
Losing	Psychomotor	3.03	1.413	1.49	0.784
	Cooperation	3.28	1.297	1.25	0.490
	Opposition	3.52	1.300	1.17	0.468
	Cooperation-opposition	3.46	1.355	1.31	0.583
Non-competitive	Psychomotor	3.59	1.202	1.24	0.506
	Cooperation	3.78	1.100	1.14	0.399
	Opposition	3.69	1.207	1.14	0.334
	Cooperation-opposition	3.83	1.124	1.11	0.308

**FIGURE 2:**
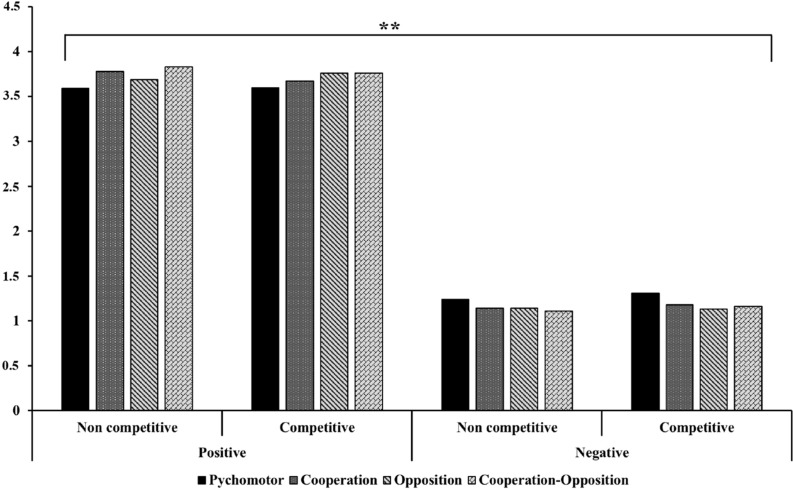
Positive and negative emotional intensity according to type of game (competitive, non-competitive) and motor action domain. Data as shown as mean. **p < 0.01.

Significant differences were observed when comparing the scores obtained for positive and negative emotions in competitive (ES = 0.946) and non-competitive games (ES = 0.956) (*p* < 0.05). In competitive games, the mean value of positive emotions was 3.7, while for negative emotions it was 1.16 (*p* = 0.000). In non-competitive games, positive emotions were experienced with a mean value of 3.69, while negative emotions it was 1.2 (*p* = 0.000). However, significant differences not were observed when comparing the score obtained for positive emotions between competitive (*M* = 3.70, SD = 0.950) and non-competitive games (*M* = 3.72, SD = 0.918, *p* = 0.618, ES = 0.02). Similarly, negative emotions not were experienced with higher scores in competitive games (*M* = 1.20, SD = 0.310) than in non-competitive games (*M* = 1.16, SD = 0.240, *p* = 0.053, ES = 0.14) (Figure 2).

### Type of Emotion X Sport Experience

Regarding the Type of emotion X Sport experience interaction, Student’s *t*-tests for independent samples revealed no significant differences in positive and negative emotions, regardless of whether or not the students performed extracurricular physical activity. However, when extracurricular physical activity was not practiced, children’s emotional intensity was greater both for positive (*M* = 3.86, SD = 0.962, *p* = 0.167, *d* = 0.24) and negative emotions (*M* = 1.22, SD = 0.296, *p* = 0.169, *d* = 0.23), whereas children who did practice extracurricular physical activity obtained lower scores both in positive (*M* = 3.64, SD = 0.856, *p* = 0.167) and negative emotions (*M* = 1.16, SD = 0.229, *p* = 0.169).

### Content Analysis

**FIGURE 3:**
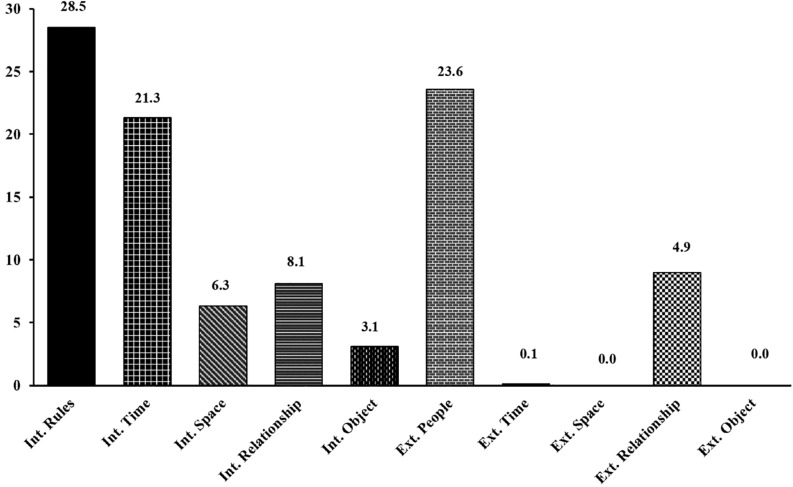
First-level content analysis of game comments. Data as shown as percentage.

Of the 1578 comments, 67.4% were related to internal aspects of the game (internal logic) and 32.6% to external aspects (external logic). The statistical analysis of the 10 micro-categories of internal and external logic showed that the rules were the most frequently mentioned variable, although people (23.6%) and internal time (21.3%) were also frequently mentioned in the comments (Figure 3).

**FIGURE 4:**
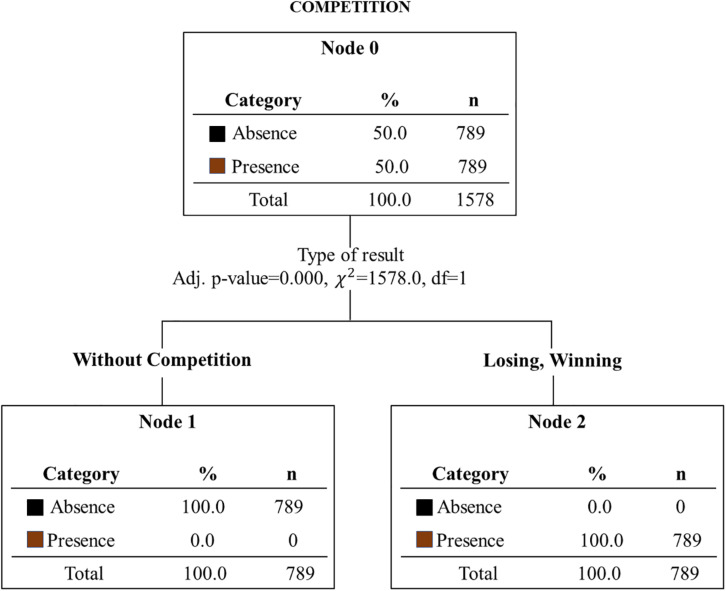
Internal time comments classification tree. Adj. *p*–value, adjusted *p*-value; χ^2^, Chi-square; df, degrees of freedom; %, percentage; *n*, number.

The subsequent statistical application of classification trees examined the predictive power of gender-independent variables, type of outcome, type of motor action domain and sport experience to predict comments related to internal time. We found that the outcome (winning, losing, and not competing) was the only explanatory factor of comments related to internal time (Figure 4).

Comments about positive emotions were more frequent and alluded to aspects of “*game rules*,” “*transient states*,” and “*internal time*,” whereas negative emotions were present in comments concerning “*internal time*,” “*transient states*,” and “*internal relationship*.”

## Discussion

Through a mixed-method approach that includes quantitative and qualitative analyses, we fulfilled the main objectives of the present study of: (a) to analyze the intensity of the emotions (positive or negative) experienced by the participants in different domains of motor action (psychomotor, cooperation, opposition, and cooperation-opposition); in non-competitive games and when winning or losing in competitive games; with or without sport experience and (b) to examine players’ comments about the most intense emotions experienced in the four motor action domains. The results obtained in the two types of analyses were compared with those of previous studies (Puig and Vilanova, 2011; Miralles et al., 2017) to determine how players interpret their emotional experience during the game. The data may differ in other social or cultural contexts.

### Emotional Experience in Relation to the Domains of Motor Action

Quantitative analysis showed a significant relationship between the emotions experienced and the game, according to its nature. The results confirm that games can be an ideal resource to teach within a motivational and pleasant context (Parlebas, 2001; Light, 2008; Etxebeste, 2012). During the different sessions, positive emotions obtained intensity values that were significantly higher than those of negative emotions, as reported in previous studies with university students (Lavega et al., 2014), secondary students (Duran and Costes, 2018), and elementary students (Howard et al., 2017; Cañabate et al., 2018). Affect is a fundamental aspect in the teaching-learning process (Parlebas, 2001) because learning depends on emotions, and the experience of positive emotions will increase motivation to learn (MacPhail et al., 2008). Although we must be aware that experiencing negative emotions is as necessary for the process of human formation than positive emotions. Teaching schoolchildren from an early age to be aware of the negative emotion they are feeling, such as anger, frustration, fear, or shame, and learn to regulate it effectively, will allow them to improve their general emotional state in adult life (Goleman, 2013).

When examining the domains of motor action, we can state that the psychomotor games and the sociomotor games of cooperation, opposition, and cooperation-opposition both generated positive emotions, with no significant differences in the players’ emotional intensities. Nonetheless, the psychomotor domain had a lower mean than the sociomotor domains, as in other studies (Lavega et al., 2014; Duran and Costes, 2018). This may be due to the fact that sociomotor games implicitly provide a series of social relationships among the players, such as negotiations, pacts, or even common challenges, that are close to the social reality, thus promoting an ideal environment for feeling positive emotions with greater intensity (Desivilya and Yagil, 2005; Helmsen et al., 2012).

The results show that, within the sociomotor games, despite the absence of significant differences between the three domains of motor action, the cooperation-opposition games generated a higher positive emotional intensity, followed by the opposition and cooperation games. Perhaps due to the age of the study participants, since they are in a stage of psycho-evolutionary development focused on continuous observation and comparison with the characteristics of the other (Blakemore, 2010). Although in similar studies (Lavega et al., 2014; Miralles et al., 2017), the cooperation domain generated more positive emotional intensity compared to the domains of opposition or cooperation-opposition. This fact, together with the results obtained in the present study, confirms the key role played by social interaction in intense emotional experience (Frijda, 2007; Diener et al., 2009). Emotional intelligence cannot be contemplated without social intelligence (Laborde et al., 2015). In fact, play is considered an emotional and social learning laboratory (Parlebas, 2001; Lagardera and Lavega, 2003).

However, negative emotions did obtain significantly different values between motor action domains. The highest negative emotional intensity was experienced in the psychomotor domain, unlike other studies (Lavega et al., 2014; Miralles et al., 2017; Duran and Costes, 2018) that reported that the most intense negative emotions were generated in sociomotor games. The key to this emotional experience may be the individual participation of the psychomotor games, as at early ages, children are accustomed to situations that require greater social participation with others (Shahid et al., 2008; Alcaraz-Muñoz et al., 2017). Therefore, psychomotor games can generate children’s emotional discomfort, making them feel embarrassed or afraid of making mistakes or that other classmates will make fun of them, sadness, feeling rejection, or anger in situations of frustration because of their lack of skill. The studies of traditional children’s games in Spain indicate that non-competitive psychomotor games are much less common than sociomotor games (Etxebeste, 2012). The fact that elementary school children experience negative emotions with greater intensity in the psychomotor domain is interesting as a future perspective to be studied more deeply. The purpose will be to know in more detail to what this experience is due, and to contrast the information with other studies where the rating of negative emotions is higher for sociomotor games.

In the sociomotor domains, the most intense rating of negative emotions was experienced in the cooperation games, as in the study with schoolchildren of Miralles et al. (2017). When a player makes a mistake during a game, the feeling of guilt is greater than in opposition or cooperation-opposition games, because in these games, there is a motor interaction with adversaries that relativizes those emotions of discomfort in the face of failures (Shahid et al., 2008; Zysberg and Hemmel, 2017). However, these results do not coincide with those obtained in the studies with university and high school students of Lavega et al. (2014) and Duran and Costes (2018). These studies reported that the negative emotional intensity increased in proportion to the relational complexity of the game, first in opposition games, where players interact with adversaries, and secondly, in cooperation-opposition games, which adds the motor relationship with a partner. In both these domains, the adversary is present, generating a greater sense of emotional discomfort than if one cannot win due to one’s own inability. However, the key to these differences may be the participants’ age, because, as age increases along with their psycho-evolutional development, children tend to pay more attention to the characteristics of the other players and even to compare themselves with them (Howard et al., 2017). These results suggest that, at early ages, when teachers wish to improve their students’ interpersonal and intrapersonal relationships, they should consider cooperation-opposition games before cooperation games (Fredrickson, 2001; Waugh and Fredrickson, 2006). If players make mistakes in cooperation games, peer rejection and their own sense of guilt is greater, unlike in cooperation-opposition games, where these feelings are relativized.

### Emotional Experience in Non-competitive Games or in the Presence or Absence of Competition

Both the quantitative and the qualitative analyses have shown that the variable “type of outcome” is a key aspect of emotional experience. The emotions felt vary depending on whether the player wins or loses. When winning, positive emotions are rated with higher intensity, whereas negative emotions obtain lower ratings. On the contrary, when losing, negative emotions obtain higher intensity scores, and positive emotions obtain lower scores. In non-competitive games, the pattern of outcomes for positive emotions resembles that observed in games that are won, although with lower the intensity ratings. On the contrary, the intensity ratings for negative emotions remain low, even lower when winning. It seems that the competitive factor increases players’ interest and levels of satisfaction compared to non-competitive games (Chen and Darst, 2001; Serna et al., 2017). These results do not coincide with previous research (Lavega et al., 2014), which indicated that non-competitive games maintained the same pattern of scores for positive emotions as lost games, although in the case of negative emotions, the values were not as high as when losing, like in the present study. These results suggest that non-competitive games are more suitable for Physical Education classes when the children have serious difficulties performing motor skills, as children will always experience negative emotions with low intensity and positive emotions with similar patterns to motor situations when winning. The way in which Physical Education teachers structure competitive motor situations directly affects the experience of the students (Harvey and O’Donovan, 2013). It is necessary to convey to teachers the importance of making appropriate use of competition in Physical Education classes, in order to teach students to learn through competition, and not only in a competitive environment. It is as necessary to teach through games with competition as without competition, to learn and experience a variety of motor and emotional situations.

### Emotional Experiences and Sport Experience

Emotional experience (positive or negative) in young players is not significantly influenced by the practice of extracurricular physical activity, unlike the case with adolescents and university students (Gea et al., 2016). The main difference between young children and adolescents or university students may be due to the fact that, although children perform physical activity regularly, they have not been doing it for enough time for it to have created a sport experience or pattern in them. This sport pattern in adolescents and university students generates significant differences in emotional intensities (positive and negative), and these intensities are lower in players with sport experience. This shows the importance of learning emotion regulation when practicing competitive, physical activity regularly in daily life. It is about learning to manage anger when losing a decisive match, the joy of scoring a goal and sharing it with one’s teammates, the fear of not performing a long jump correctly. These individuals are constantly accustomed to this type of situations with a high level of demand and emotional commitment. According Lazarus (1991), people with and without sport experience behave and react differently because their emotions are organized hierarchically, such that the greater the importance of the objective to be reached, the greater will be the emotional intensity experienced. Players with greater sport experience grant less importance to motor situations in the educational area, because the objective is less relevant than the goal they have set for the season at the competitive level. These results show the need for teachers to transmit to their students the importance of practicing physical activity regularly throughout life, to promote not only active people but also emotionally intelligent people (Frijda, 2007; Bailey et al., 2009). In addition, physical education teachers are advised to know the sports profile of students, since it can be a variable that with their development can favorably influence the emotional experience of schoolchildren, when they participate in traditional sporting games.

## Conclusion

Based on the results obtained, we can state that these physical education sessions are presented as a motivating learning climate for students, generating high values of positive emotional intensity independently of the motor domain, emphasizing games of cooperation-opposition, opposition, and cooperation. On another hand, the experience of negative emotions is also present, although with lower values, with the psychomotor domain generating a significantly higher intensity. These results are evidence to be considered by Physical Education teachers to determine their pedagogical practices aimed at minimizing the experience of negative emotions (Puig and Vilanova, 2011). However, when Physical Education teachers consider competitive or non-competitive games in their classes, they must also pay attention to the experience of emotions depending on their outcome. When players win, they feel considerable positive emotion, unlike in non-competitive games, which also allow them to experience emotional well-being, but with lower values. However, when players lose in competitive games, they feel negative emotions with a greater intensity, different from any non-competitive or competitive situation where they win.

Games seek the student’s motor, social, cognitive, and emotional development, and the study of emotions is a key aspect. However, given the multidimensional and complex nature, not only of motor skills (Parlebas, 2001; Etxebeste, 2012) but also of emotions, social, and cognitive (Lazarus, 1991; Bisquerra, 2003; Frijda, 2007; Light, 2008), it is important to consider the findings obtained in this article as an initial contribution for early ages, which require more interdisciplinary studies.

## Data Availability Statement

The original contributions presented in the study are included in the article/supplementary materials, further inquiries can be directed to the corresponding author.

## Ethics Statement

The studies involving human participants were reviewed and approved by The University Ethics Committee of the Murcia University (UM) reviewed and approved the research in accordance with the principles set out in the Declaration of Helsinki (Code: 1684/2017). Written informed consent to participate in this study was provided by the participants’ legal guardian/next of kin.

## Author Contributions

VA-M, JA, and JY conceptualized and designed the study. VA-M and MC recruited the subjects. VA-M, MC, JA, and JY collected the data. JY and VA-M organized the database and carried out the statistical analysis. VA-M, JA, JY, MC, and GG wrote the first manuscript draft. JA and GG developed the final manuscript draft, the English proofreading and, reviewed and edited the final version of the manuscript. All authors contributed to the manuscript revision and approved the definitive manuscript.

## Conflict of Interest

The authors declare that the research was conducted in the absence of any commercial or financial relationships that could be construed as a potential conflict of interest.
